# Supplementary material for: Chromothripsis during telomere crisis is independent of NHEJ, and consistent with a replicative origin
Source: Genome Res. 2019 May;29(5):737–49. doi: 10.1101/gr.240705.118 (PMC6499312; doi:10.1101/gr.240705.118)
Supplement: Supplemental Material [file supp_gr.240705.118_Supplemental_file_1.zip › contigs/annotated_contigs/DB82/contig.2.DB82_length_698_mean_cov_6.12607449857.docx]

**DB82_length_698_mean_cov_6.12607449857**

```
GACTTGGTCTGTTTGAGGTTTACTATTCAATGGAAGCATATCTAAGTCACTTAGCATCAGCTCACTCTACATGCCCTCAACTATTGAAA
 >chr5:13310250-13310614 - E=3e-207 p=3e-03
GGCACCTTCATCTTCACATTTTTCAAGTAGGAGGGAAAGAGGATGTGAACAGCCCTCACAATGAAGAGGCGAAAACTTGTCTGAAAGGT

AGAAAGCTGGCATTGCCAACTGGTTAATTGAAGCTTGAATGCCAAGTTTCAAGTTCTTGAAGGTTAAGAAAGCCTTTCTTATTGGTTAA

TAGGGAGATAAGTGAGGATCATCCTCGAGGCATCTCAAAATCTGTGCTCATCGGCCATGTCCTCCTGAACTTTTGCCTCTGAGGGAACA

CAAATAGT|ATATATACTA|GGGTATCCTACAGAAGCCAGGGCTCTTTGTCATAAAGCTAAACACACACACACATGGTCTCAAAGTTGG
                   >chr5:13308024-13308348 - E=4e-180
AGAAGCCAAAGTAGAATCCAGGGAAAGAAGAAGCAGTGGCAGCCTTGGTTGTTTTTCATACCAGTGATGTGAAACAACAGATCGGCAAC

ATTCTGTGTGAGCCAAAAAGTCTGGTGCTTCGTGTCCACCCTCCAAATCTTGCATAGAAATATCTTTTGTGGCTCACTTTAACAGAAAC

ATATGGTATCCATCAGAGGACAATCAAATATAGTCAATGTCAAAACTTTTGCCTCAGATCATCCCGAGGCTTTGAGT
```
